# Supplementary material for: Influence of Tea Consumption on the Development of Second Esophageal Neoplasm in Patients with Head and Neck Cancer
Source: Cancers (Basel). 2019 Mar 19;11(3):387. doi: 10.3390/cancers11030387 (PMC6468666; doi:10.3390/cancers11030387)
Supplement: Supplementary file 1 [file cancers-11-00387-s001.pdf]

Supplementary Materials: Influence of Tea Consumption on the Development of Second Esophageal Neoplasm in Patients with Head and Neck Cancer

Yao-Kuang Wang, Wei-Chung Chen, Ying-Ho Lai, Yi-Hsun Chen, Ming-Tsang Wu, Chie-Tong Kuo, Yen-Yun Wang, Shyng-Shiou F. Yuan, Yu-Peng Liu and I-Chen Wu

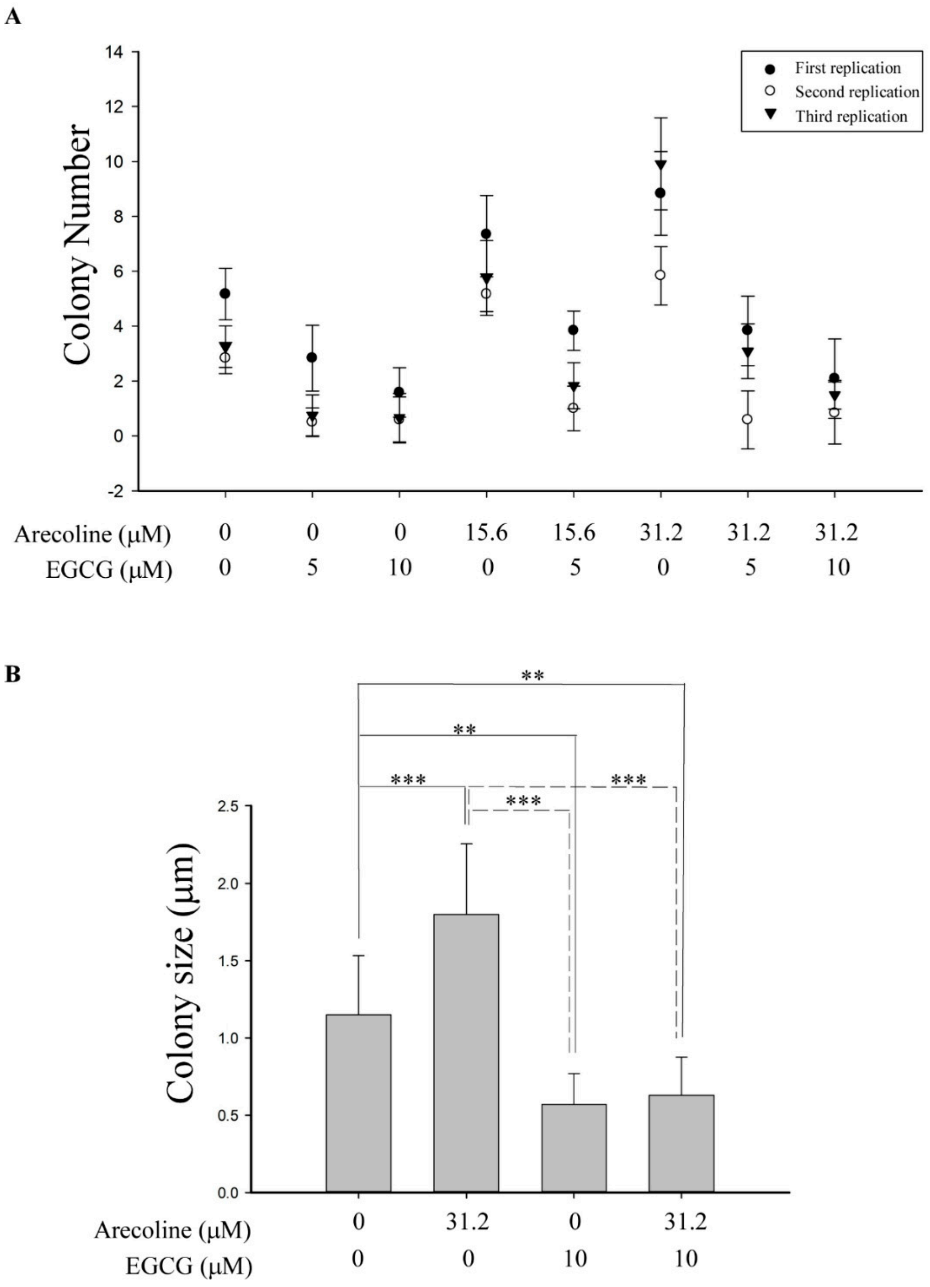

Figure S1. EGCG reduced the numbers and size of arecoline induced cell colonies. (**A**) Triple replications of colony formation assay of Figure 1D. Each group in single replication contained twelve repeat measurements. (**B**) Colony size of triple replications in Figure 1D. ** $p < 0.01$; *** $p < 0.001$.

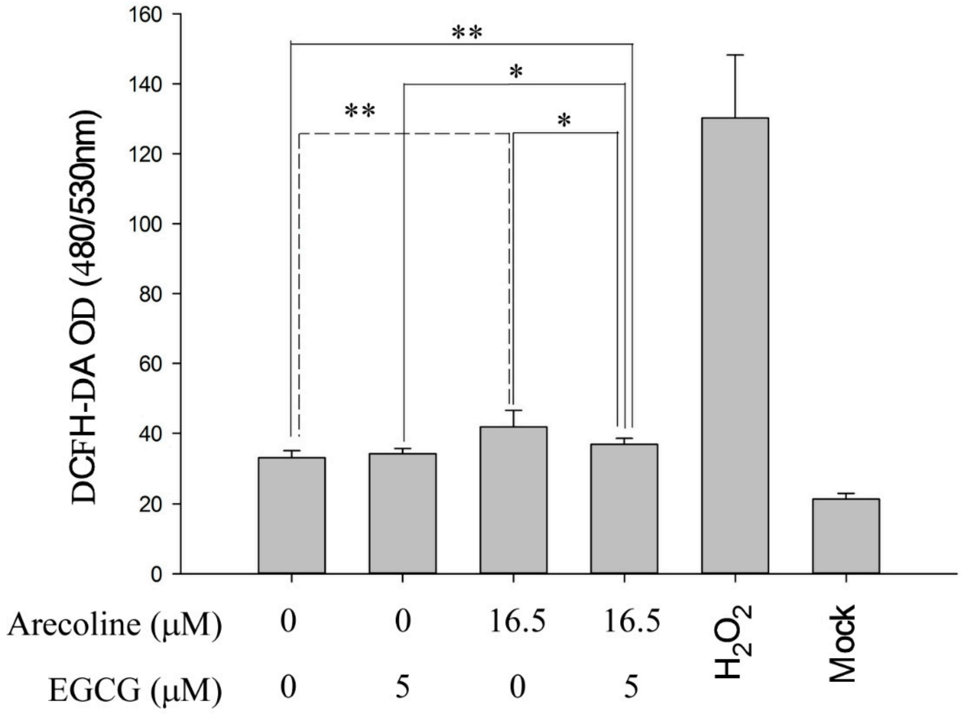

Figure S2. DCFH-DA intracellular ROS level quantification in OE21 after treating arecoline for 8 h. Each group contained six repeat measurements. * $p < 0.05$; ** $p < 0.01$.

© 2019 by the authors. Licensee MDPI, Basel, Switzerland. This article is an open access article distributed under the terms and conditions of the Creative Commons Attribution (CC BY) license (http://creativecommons.org/licenses/by/4.0/).
